# Energy Efficiency Optimization for Downlink Cloud RAN with Limited Fronthaul Capacity

**DOI:** 10.3390/s17071498

**Published:** 2017-06-26

**Authors:** Yong Wang, Lin Ma, Yubin Xu

**Affiliations:** School of Electronics and Information Engineering, Harbin Institute of Technology, Harbin 150080, China; dr_ywang@hotmail.com (Y.W.); malin@hit.edu.cn (L.M.)

**Keywords:** C-RAN, fronthaul compression, energy efficiency, alternating direction method of multipliers (ADMM), imperfect channel state information (CSI)

## Abstract

In the downlink cloud radio access network (C-RAN), fronthaul compression has been developed to combat the performance bottleneck caused by the capacity-limited fronthaul links. Nevertheless, the state-of-arts focusing on fronthaul compression for spectral efficiency improvement become questionable for energy efficiency (EE) maximization, especially for meeting its requirements of large-scale implementation. Therefore, this paper aims to develop a low-complexity algorithm with closed-form solution for the EE maximization problem in a downlink C-RAN with limited fronthaul capacity. To solve such a non-trivial problem, we first derive an optimal solution using branch-and-bound approach to provide a performance benchmark. Then, by transforming the original problem into a parametric subtractive form, we propose a low-complexity two-layer decentralized (TLD) algorithm. Specifically, a bisection search is involved in the outer layer, while in the inner layer we propose an alternating direction method of multipliers algorithm to find a closed-form solution in a parallel manner with convergence guaranteed. Simulations results demonstrate that the TLD algorithm can achieve near optimal solution, and its EE is much higher than the spectral efficiency maximization one. Furthermore, the optimal and TLD algorithms are also extended to counter the channel error. The results show that the robust algorithms can provide robust performance in the case of lacking perfect channel state information.

## 1. Introduction

To maintain the requirements of the expected scale of the increasing data traffic and mobile terminals, the fifth generation (5G) wireless network [1] faces some challenges in terms of system capacity, energy consumption, and so on. The cloud radio access network (C-RAN) [2,3], which has emerged as a promising solution in reducing both the capital and operating expenditures, is expected to be an effective approach to fulfil these requirements. In C-RAN, a central unit (CU) or baseband unit (BBU) pool connects all the deployed low-power base stations (BSs) using the finite-capacity fronthaul links that allows joint signal processing and transmission. Despite various attractive advantages brought by C-RANs [4,5,6], such as joint beamforming, and centralized encoding and decoding, the performance bottleneck for large-scale implementation comes with the high capacity requirements of the fronthaul links. Therefore, in practical C-RAN, the data sharing [7,8,9,10,11,12,13] and fronthaul compression designs [14,15,16,17,18,19,20,21,22] are recognized as two promising approaches to overcome the significant impact of the constrained fronthaul on spectral efficiency (SE) and energy efficiency (EE) (bit-per-Joule) [23,24,25]. The data sharing strategy [8,9,10,11,12,13] reduces the fronthaul consumption through limiting the data transfer among BSs (one BS serves a small number of the total MUs). For the latter one, the CU computes the precoded signals intended to be transmitted to each BS, and then the signals are quantized and sent to the BSs through the capacity limited fronthaul links. The compression process is usually modeled as a test channel [14,15,16,17,18,19,20,21,22], where the uncompressed and compressed signals respectively represent the input and output, and the quantization noise are modeled as an independent additive Gaussian random variable [13,20,21]. The authors in [13] proved that the fronthaul compression achieves a better performance than the data sharing strategy, so that this paper considers the fronthaul compression.

In general, the fronthaul compression has been extensively studied in both the uplink [16,17,18] and downlink C-RANs [13,19,20,21,22]. The majority research of the existing literature on fronthaul compression in C-RAN is focused on the design of beamforming and quantization noises to maximize the achievable sum rate from the information-theoretic perspective [11,13,16,17,18,19,20,21,26]. A joint adaptive decompression and detection algorithm was proposed in [16] to improve the information-theoretic capacity for the uplink C-RAN. In [18], the authors developed a distributed compression for the sum rate maximization (SEmax) problem in the uplink C-RAN accounting for both perfect and imperfect channel state information (CSI). For the downlink C-RAN, a joint precoding and multivariate compression scheme has been studied in [19], where an iterative majorization-minimization approach was proved to achieve a stationary point solution of the SEmax problem. In [13], a comparison between the data sharing and fronthaul compression strategy was investigated for the power minimization problem with finite-capacity fronthaul links in downlink C-RAN. Then, a hybrid compression and data sharing strategy was designed in [11] for optimizing the achievable sum rate. However, maximizing the EE in accordance with fronthaul compression [22] is technically far more challenging compared to SRmax, and the closed-form solution remains unexplored.

This paper investigates the EEmax problem in downlink C-RANs with the consideration of the fronthaul compression. This optimization problem is formulated as a non-convex fractional programming problem with respective to the beamforming and quantization noises under a limited power budget and capacity-finite fronthaul links, which is NP-hard and is difficult to solve. To deal with the fraction objective function of the original problem, some work has been done to firstly transform the fractional objective function into an equivalent subtractive-form optimization problem via exploiting the fractional programming [27,28,29,30]. Although the power allocation schemes for orthogonal frequency division multiple access (OFDMA) system [28,29] were obtained using the dual decomposition approach, such approaches cannot be applied to solve our problem due to the joint consideration of fronthaul compression and beamforming in our problem. Moreover, the Lagrangian based decomposition algorithm [30] for the multicell system is still not applicable for decentralized implementation because the considered problem is more complex and the beamformings among BSs are coupled. The first-order Taylor expansion is adopted to linearize the non-convex data rate and fronthaul capacity constraint [20], however, the optimal solution cannot be obtained. By exploiting the relationship between the achievable data rate and the mean square error (MSE) in [31], Ref. [11,12] compared the sum rate performance of the data sharing and compression strategies, but they are limited to the SRmax problem. The authors in [22] considered a joint design of beamforming, multivariate compression and BS-MU link selection to maximize the EE. By using the epigraph form of the original EEmax problem, the authors [22] proposed a difference of convex (DC) function based algorithm. However, the optimal solution of [22] is still unknown, and the computational complexity is typically high since a series of semi-definite programming (SDP) or second-order cone programming (SOCP) problems are solved. More importantly, since the beamforming and quantization noises are computed centrally at the CU, the algorithms proposed in the literature [11,12,13,19,20,22] can be computation intensive for large-scale C-RANs. Therefore, in this paper, we first propose an optimal algorithm for the EEmax problem to provide a performance benchmark, and then develop a low-complexity decentralized algorithm with closed-form solution. For practical usage in downlink C-RANs, the impact of CSI errors on the EE performance is further investigated. This paper not only offers an optimal solution for the EEmax problem with limited fronthaul capacity, but also lays a sound foundation for decentralized implementation with closed-form solutions in this field. Thus, the methods derived in this paper are significant in advancing this field.

The main contributions of this paper are summarized as follows.

*Firstly*, we formulate the EEmax problem of joint beamforming and quantization noises design under a limited power budget and capacity-limited fronthaul links as a non-convex fractional programming problem. We first derive an optimal solution method based on branch-and-bound (BnB) technique [32,33] to solve the EEmax problem globally. Specifically, the BnB algorithm computes the upper and lower bounds, and deletes the regions that do not contain the optimal solution. The algorithm terminates when the difference between the upper and lower bounds is smaller than a predefined accuracy.

*Secondly*, to reduce the computational complexity of the optimal algorithm and facilitate decentralized implementation, we propose to transform the problem into a parametric subtractive form, and further proposed a two-layer decentralized (TLD) algorithm to solve the equivalent subtractive problem. Specifically, an one-dimension search approach is used to find the EE in the outer layer, and a decentralized algorithm based on alternating direction method of multipliers (ADMM) is proposed to solve a subproblem in the inner layer. The proposed algorithm achieves closed-form solution in parallel manner with convergence guaranteed.

*Thirdly*, considering the imperfection of the obtained CSI in practical C-RANs, the robust optimal and TLD algorithms for the considered EEmax problem are also proposed to characterize the performance degradation of the CSI errors. In particular, the robust optimal can also achieve a performance benchmark, and the robust TLD algorithm also has closed-form solution in a parallel manner.

*Finally*, we validate the effectiveness of the proposed algorithms through extensive simulations. The results demonstrate that both the optimal and TLD algorithms are convergent, and the TLD algorithm can achieve near optimal solution which is much higher than the SEmax one. Numerical analysis also show that the EE performance is susceptible to the channel errors, and a smaller channel error reaches a higher EE.

The remainder of the paper is organized as follows. In Section 2, the system model and problem formulation are presented. Section 3 describes the proposed optimal algorithm with perfect CSI. Section 4 presents TLD algorithm with perfect CSI. For imperfect CSI case, the robust optimal and TLD algorithms are also presented in Section 5. The simulation results are given in Section 6. Finally, we conclude this paper in Section 7.

*Notations*: We use C to denote the set of complex numbers, and $C^{M\times N}$ to denote the set of all M×N matrices with complex entries. We use boldface capital and lower case letters are respectively used to denote matrices and vectors. ${\left(X\right)}^{-1}$, $X^{H}$ and Tr(X) represent the matrix inverse, Hermitian transport and the trace, respectively. |x| represents the Euclidean norm. E[·] is the expectation operator, and $\mathrm{diag}(x_{1},\ldots ,x_{L})$ represents a diagonal matrix with diagonal elements given by $\left\{x_{1},\ldots ,x_{L}\right\}$. For a complex number *x*, |x| is the mode of *x*. “s.t.”stands for “subject to”.

## 2. System Model and Problem Formulation

### 2.1. System Model

We consider a downlink C-RAN with *L* single-antenna BSs and *K* single-antenna MUs. The CU connects all the BSs via fronthaul links, and each link is finitely constrained by $C_{l},l=1,\ldots ,L$. Assume that the CU has access the global CSI. The data symbol for each MU (denoted by $s_{k}$ for the *k*-th MU) is distributed as complex Gaussian with zero mean and unit variance. Denote by $x_{l}=\sum_{k=1}^{K}w_{kl}s_{k}$ the beamformed complex signal at the CU for BS *l*, where $w_{kl}$ is the beamforming from BS *l* to MU *k*. To reduce the capacity requirements on the fronthaul network, the signals are compressed before being forwarded to the corresponding BSs via the finite-capacity fronthaul links. According to [18,20], the compression procedure is modeled as a test channel and the procedure can be expressed as ${\tilde{x}}_{l}=x_{l}+e_{l},\forall l$, where $e_{l}$ is the quantization noise, and $x_{l}$ and ${\tilde{x}}_{l}$ are the input and out of the test channel, respectively. The received signal at MU *k* is given by

(1) $$y_{k}=\sum_{l=1}^{L}h_{kl}^{H}{\tilde{x}}_{l}+n_{k},k=1,\ldots ,K,$$

where $h_{kl}\in C$ is the channel from BS *l* to MU *k*, $n_{k}$ is the additive Gaussian noise at MU *k*, with zero mean and $\sigma^{2}$ variance.

When employing the single user detection at each MU, the received signal-to-interference-plus-noise-ratio (SINR) at MU *k* is

(2) $$\begin{array}{cc} {\mathrm{SINR}}_{k} & =\frac{|h_{k}^{H}w_{k}{|}^{2}}{\sum_{i\neq k}|h_{k}^{H}w_{i}{|}^{2}+\sigma^{2}+\left|h_{k}Qh_{k}^{H}\right|}, \end{array}$$

where $h_{k}={\left[h_{k1}^{T},\ldots ,h_{kL}^{T}\right]}^{T}$ and $w_{k}={\left[w_{k1},\ldots ,w_{kL}\right]}^{T}$ are the aggregated channel and beamforming from all BSs to MU *k*, respectively, and

(3) $$\begin{array}{c} Q=\left[\begin{array}{ccc} q_{11} & \ldots & q_{1L}\\ \vdots & \ddots & \vdots \\ q_{L1} & \ldots & q_{LL} \end{array}\right], \end{array}$$

is the covariance matrice of $e={\left[e_{1},\ldots ,e_{L}\right]}^{T}$, i.e., $Q=ee^{H}$. It is noted that multivariate compression is also possible and has been studied in [20], where $e_{l}e_{j}^{H}\neq 0,\forall l=1,\ldots ,L$., $e_{l}e_{j}^{H}=0$ when l≠j. In this paper, we consider point to point fronthaul compression, and let $q_{l}=q_{ll},l=1,\ldots ,L$.

Considering an ideal vector quantizer, the quantization noise level $q_{l}$ and the fronthaul capacity $C_{l}$ for the *l*-th fronthaul link satisfy the following constraint [12,15]

(4) $$\log_{2}\left(1+\frac{\sum_{k=1}^{K}{\left|w_{kl}\right|}^{2}}{q_{l}}\right)\leq C_{l},\forall l.$$

The transmission power consumed at BS *l* is constrained by $E[|{\tilde{x}}_{l}{|}^{2}]\leq P_{l}^{\max}$, where $P_{l}^{\max}$ is the maximum transmit power of BS *l*. The transmit power of BS *l*, denoted by $p_{l}$, consists of quantization noise $q_{l}$ and data transmission power (denoted by $p_{l}^{t}$), i.e., $p_{l}=p_{l}^{t}+q_{l}=\sum_{k=1}^{K}{\left|w_{kl}\right|}^{2}+q_{l}$. It is obviously that

(5) $$p_{l}\leq P_{l}^{\max},\forall l.$$

The network power of C-RAN consists of the BS transmit power and relative fronthaul network power. In this paper, we adopt the power consumption model of C-RAN as [4]

(6) $$P_{tot}=\sum_{l=1}^{L}\frac{1}{\eta_{l}}p_{l}+P^{c},$$

where $P^{c}=\sum_{l=1}^{L}P_{l}^{c}$ is the total relative fronthaul link power consumption [4], $P_{l}^{c}\geq 0$ is the relative fronthaul link power consumption when switch off both the fronthaul link and the corresponding BSs. $\eta_{l}$ ($\eta_{l}>1$) is the drain efficiency of power amplifier of BS *l*. In this paper, we assume that all the BSs have the same drain efficiencies, i.e., $\eta =\eta_{l},\forall l$. Since we do not consider the BS switch on/off scheme in this paper, $P^{c}$ is a nonnegative constant and we call it by static power for brevity for the rest of the paper. We point out that based on the results obtained by the proposed algorithms in this paper, it is easily extended to add BSs selection (determine the BSs to be switch off or not) into consideration through ordering the BSs in accordance with the bisection search [4] to further improve EE. However, this is outside the scope of this paper.

### 2.2. Problem Formulation

To balance the sum rate and total power consumption, the EEmax problem is optimized in this paper. This problem over the beamforming in the presence of fronthaul compression can be formulated as

(7a) $$\begin{array}{cc} P_{0}:\;\max_{w,q} & \;\frac{\sum_{k=1}^{K}R_{k}}{\sum_{l=1}^{L}\frac{1}{\eta_{l}}p_{l}+P^{c}} \end{array}$$

(7b) $$\begin{array}{cc} s.t. & \;\sum_{k=1}^{K}{\left|w_{kl}\right|}^{2}+q_{l}\leq P_{l}^{\max},\forall l \end{array}$$

(7c) $$\begin{array}{c} \;\beta_{l}\sum_{k=1}^{K}{\left|w_{kl}\right|}^{2}\leq q_{l},\forall l, \end{array}$$

where $w=[w_{1},\ldots ,w_{K}]$ and $q=[q_{1},\ldots ,q_{K}]$ are the collection of beamforming vectors and quantization noises, respectively. $R_{k}=\log_{2}\left(1+{\mathrm{SINR}}_{k}\right)$ is the achievable data rate of MU *k*. (7b) is the BS transmit power constraint, and (7c) is the reformulation of the fronthaul capacity constraint (4) and $\beta_{l}=\frac{1}{2^{C_{l}}-1}$. Due to the non-convex $R_{k}$ and the fractional objective function in (7a), $P_{0}$ is a NP-hard problem, and it is challenging to find its global optimum. In the following, we first present an optimal approach and then propose a TLD framework solution.

## 3. Optimal Algorithm with Perfect CSI

In this section, we will propose a global optimal algorithm, which based on Branch-and-Bound method [32], to solve problem $P_{0}$. The essential idea of the proposed algorithm is based on the following equivalent transformation

(8a) $$\begin{array}{cc} P_{1}:\max_{w_{kl},t} & \;f\left(t\right)=t_{K+1}\sum_{k=1}^{K}t_{k} \end{array}$$

(8b) $$\begin{array}{cc} s.t. & \;{\mathrm{SINR}}_{k}\geq 2^{t_{k}}-1,\forall k \end{array}$$

(8c) $$\begin{array}{c} \;\frac{1}{\frac{1}{\eta }\sum_{l=1}^{L}(\sum_{k=1}^{K}|w_{kl}{|}^{2}+q_{l})+P_{c}}\geq t_{K+1}\\ \;(7b),(7c), \end{array}$$

where $t={\left[t_{1},\ldots ,t_{K+1}\right]}^{T}$ are the introduced variables, (8b) is the transformation of $\log_{2}\left(1+{\mathrm{SINR}}_{k}\right)\geq t_{k}$. The equivalence between problems $P_{0}$ and $P_{1}$ is that the constraints (8b) and (8c) hold with equality at optimum.

Although $P_{1}$ is more tractable compared to $P_{0}$, it is still hard to solve due to the coupled variables of $w_{k}$ and t. It is observed that if one increase each $t_{k}$ in the feasible set of $P_{1}$, a better objective value can be obtained. This motivates us to use the monotonic optimization in [32], i.e., the optimal BnB algorithm, to solve problem $P_{1}$.

### BnB Algorithm

To solve $P_{1}$, we first denote the feasible set for variables t by Ξ, i.e., $\Xi =\{t|\mathrm{constraints of}\;P_{1}\}$. Denote $\underline{t}={\left[{\underline{t}}_{1},\ldots ,{\underline{t}}_{K+1}\right]}^{T}$ and $\bar{t}={\left[{\bar{t}}_{1},\ldots ,{\bar{t}}_{K+1}\right]}^{T}$ by the aggregated lower and upper bound of $t_{k}$. The interval $\underline{t}\leq t\leq \bar{t}$ indicates that each element of t is bounded by its lower and upper bounds. The objective function f(t) in $P_{1}$ is monotonically increasing in the interval $\underline{t}\leq t\leq \bar{t}$. In particular, $t_{k}$ is upper bounded by ignoring the interferences, i.e., $t_{k}\leq \log(1+\frac{1}{\sigma_{k}^{2}}\sum_{l=1}^{L}P_{l}^{m}|{\tilde{h}}_{k}{|}^{2})={\bar{t}}_{k}$, and the lower bound of $t_{k}$ is ${\underline{t}}_{k}=0\leq t_{k}$ for k=1,…,K. Similarly, we can constrain $t_{K+1}$ by ${\underline{t}}_{K+1}\leq t_{K+1}\leq {\bar{t}}_{K+1}$, where ${\bar{t}}_{K+1}=1/P_{c}$ and ${\underline{t}}_{K+1}=\frac{1}{\sum_{l=1}^{L}P_{l}^{m}/\eta +P_{c}}$. It is obvious that the feasible set t in Ξ must be contained by $\Phi =[\underline{t},\bar{t}]$.

For a given $\underline{t}\in \Phi$, problem $P_{1}$ reduces to the feasibility problem given by

(9) $$\begin{array}{cc} P_{2}:\mathrm{find} & \;w_{1},\ldots ,w_{K},q_{l}\\ s.t. & \;(7b),(7c),(8b),(8c). \end{array}$$

Obviously, when $\underline{t}={\left[0,0,\ldots ,\frac{1}{\sum_{l=1}^{L}P_{l}^{m}/\eta +P_{c}}\right]}^{T}$, problem $P_{2}$ is infeasible. That is because the sum rate should not equal to zero with maximum transmit power. In this paper, we will customize the BnB algorithm to solve problem $P_{1}$ globally. The BnB algorithm divides the box Φ into smaller ones, and cuts off boxes that do not contain an optimal solution. The algorithm will converge to the global optimal solution after finite iterations.

Since $P_{2}$ is non-convex due to the SINR constraint, in the following, we first recast them as convex ones. Let ${\tilde{\gamma }}_{k}=2^{t_{k}}-1$, (8b) is equivalently rewritten as [7,33]

(10a) $$\begin{array}{c} \frac{1}{{\tilde{\gamma }}_{k}}h_{k}w_{k}\geq {\left(\sum_{i\neq k}|h_{k}^{H}w_{i}{|}^{2}+\sigma^{2}+\sum_{l=1}^{L}q_{l}{\left|h_{kl}\right|}^{2}\right)}^{1/2} \end{array}$$

(10b) Im(hkwk)=0,∀k∈K.

In the above formulation, we note that (10a) is a second-order cone (SOC) constraint. The constraint (10b) is without loss of generality due to the fact that a phase rotation of the beamformers does not effect the objective of the problem [25,33].

Moreover, (8c) is easily rewritten as

(11) $$\frac{1}{\eta }\sum_{l=1}^{L}\left(\sum_{k=1}^{K}{\left|w_{kl}\right|}^{2}+q_{l}\right)\leq \frac{1}{t_{K+1}}-P_{c}.$$

Then, problem $P_{2}$ becomes a SOCP feasibility problem which can solved efficiently. In the proposed BnB algorithm, the bounding function can be formally expressed as

(12) $$\phi_{\mathrm{ub}}\left(\Phi \right)=\left\{\begin{array}{cc} f(t_{\max}), & t_{\min}\in \Xi \\ 0, & \mathrm{otherwise}, \end{array}\right.$$

(13) $$\phi_{\mathrm{lb}}\left(\Phi \right)=\left\{\begin{array}{cc} f(t_{\min}), & t_{\min}\in \Xi \\ 0, & \mathrm{otherwise}, \end{array}\right.$$

where $\phi_{\mathrm{ub}}\left(\Phi \right)$ and $\phi_{\mathrm{lb}}\left(\Phi \right)$ are the upper and lower bound respectively, Φ is defined as $\Phi ≜\{t|t_{k,\min}\leq t_{k}\leq t_{k,\max},\forall k\}$ where $t_{k,\min}$ and $t_{k,\max}$ denote the end points of the *k*th edge of Φ, $t_{k,\min}={\left[t_{1,\min},\ldots ,t_{K+1,\min}\right]}^{T}$ and $t_{k,\max}={\left[t_{1,\max},\ldots ,t_{K+1,\max}\right]}^{T}$.

We denote by $V_{i}$ the collection of all created boxes at iteration *i*. Then, the work flow of the BnB algorithm to obtain the global optimal solution is presented in Algorithm 1.

**Algorithm 1** Proposed Optimal Algorithm. 0: Initialization: given tolerance τ>0. Set i=1, $B_{1}=\left\{\Phi \right\}$, $U_{1}=\phi_{\mathrm{ub}}\left(\Phi \right)$, $L_{1}=\phi_{\mathrm{lb}}\left(\Phi \right)$. 1: Check the feasibility problem $P_{2}$ with given $\underline{t}$. If it is infeasible, exit; Otherwise go to step **2**. 2: **repeat** 3:    Set $\Phi_{i}=\Phi$ where Φ satisfies $U_{i}=\phi_{\mathrm{ub}}\left(\Phi \right)$. 4:    Branch $\Phi_{i}$ into two smaller boxes $\Phi_{I}$ and $\Phi_{II}$ using the bisection subdivision along the longest edge of $\Phi_{i}$. 5:    Let $B_{i+1}=\left(B_{i}\backslash \left\{\Phi_{i}\right\}\right)\cup \left\{\Phi_{I},\Phi_{II}\right\}$. 6:    Update $L_{i+1}=\max_{\Phi \in B_{i+1}}\left\{\phi_{\mathrm{lb}}\left(\Phi \right)\right\}$. 7:    Delete boxes that do not contain an optimal solution $V_{i+1}=V_{i}\backslash \left\{\Phi_{k}|L_{i+1}>\phi_{\mathrm{ub}}\left(\Phi_{k}\right)\right\}$ where $\Phi_{k}\in B_{i+1}$. 8:    Update $U_{i+1}=\max_{\Phi \in V_{i+1}}\left\{\phi_{\mathrm{ub}}\left(\Phi \right)\right\}$. 9:    Set i=i+1. 10: **Until**
$U_{i+1}-L_{i+1}\leq \tau$.

**Remark** **1** (H)**.** According to [34], the convergence of Algorithm 1 is guaranteed due to the monotonic property of f(t). The main step of Algorithm 1 is to delete the boxes that do not contain the optimal solution. This step is referred to as pruning, and a smaller box that contains the optimal solution is obtained. Therefore, step 7 confirms the convergence of Algorithm 1. The corresponding optimal EE is $f{\left(t\right)}^{⋆}=U_{i}$, the optimal achieved data rate is $t^{⋆},\forall k\in K$, and the network power consumption is $1/t_{K+1}^{⋆}$. This algorithm gives an optimal solution to problem $P_{1}$ (equivalently to problem $P_{0}$) when the tolerance τ is small enough. Algorithm 1 provides a performance benchmark for any other suboptimal algorithms. However, the computational complexity of Algorithm 1 is very high in general. Therefore, an improved box reduction approach approach was proposed in [33,34] to reduce the searching time, but we use the basic BnB approach in this paper for simplicity.

## 4. Decentralized Algorithm with Perfect CSI

In this section, we first transform the original problem into an equivalent subtractive-form using the Dinkelbach’s method. By exploiting the equivalence between the achievable data rate and its MSE, an ADMM algorithm is proposed to solve a QCQP subproblem with closed-form solution in a parallel manner.

### 4.1. Equivalent Optimization Problem

It is noted that $P_{0}$ is a nonlinear fractional programming problem and can be transformed using the Dinkelbach’s method [27]. Defining the optimal EE of problem $P_{0}$ by $\alpha^{\mathrm{opt}}$, we have

(14) $$\alpha^{\mathrm{opt}}=R^{⋆}/P_{tot}^{\mathrm{opt}}=\max_{\left\{w,q\right\}}R/P_{tot},$$

where $P_{tot}^{\mathrm{opt}}$ is the optimal total power consumption, $R^{\mathrm{opt}}$ is the optimum of *R*, and $R^{\mathrm{opt}}=\sum_{k=1}^{K}R_{k}^{\mathrm{opt}}$ is the sum rate.

According to [29], the optimal EE $\alpha^{\mathrm{opt}}$ can be achieved if and only if

(15) $$\max_{w,q}\sum_{k=1}^{K}R_{k}-\alpha^{\mathrm{opt}}P_{tot}=R^{\mathrm{opt}}-\alpha^{\mathrm{opt}}P_{tot}^{\mathrm{opt}}=0,$$

where (w,q)∈D and D={(w,q)|(7b),(7c)} is the feasible region of problem $\alpha^{\mathrm{opt}}$.

Thus, based on the theoretical results in [25,30,33], problem $P_{0}$ is transformed as the following parametric programming problem

(16) $$\begin{array}{cc} P_{3}:\;G\left(\alpha \right)= & \max_{w,q}\;\sum_{k=1}^{K}R_{k}-\alpha P_{tot}\\ & s.t.\;(7b),(7c), \end{array}$$

It is noted that if problem $P_{3}$ is optimally solved with G(α)=0, problem $P_{0}$ can be solved optimally. However, if problem $P_{3}$ cannot be optimally solved, we can still solve problem $P_{0}$ through solving a sequence of problem $P_{3}$. Unfortunately, the optimal solution of problem $P_{0}$ is not guaranteed in this case. We will provide detailed analysis in the next subsection.

The function of G(α) is a monotonically decreasing function over α. Therefore, a bisection method, which is demonstrated in Algorithm 2, should perform well enough to find α [25,30].

**Algorithm 2** Outer Layer Algorithm. 1: Initialize the minimum and maximum α as $\alpha_{\min}$ and $\alpha_{\max}$ respectively, and a small threshold value ϵ. 2: **repeat** 3:    Set $\alpha =(\alpha_{\max}+\alpha_{\min})/2$, solve problem $P_{3}$ with α. 4:    If G(α)≥0, $\alpha_{\min}=\alpha$. Otherwise, $\alpha_{\max}=\alpha$. 5: **Until**
$|\alpha_{\max}-\alpha_{\min}|\leq \epsilon$ or the maximum iteration number is reached.

It is important to initialize α in reducing the search time of Algorithm 2. Here, we initialize the interval $\alpha_{\min}\leq \alpha \leq \alpha_{\max}$ that α is bounded by its lower and upper bounds. Intuitively, α is lower bounded by $\alpha_{\min}=0$ when the the sum rate equals to zero. For $\alpha_{\max}$, it is upper bounded by ignoring the interference and using maximum transmit power in $R_{k}$, and ignoring the transmit power and the quantization noises in $P_{tot}$. Specifically, $R_{k}\leq \log_{2}(1+\frac{1}{\sigma^{2}}\sum_{l=1}^{L}P_{l}^{\max}|h_{k}{|}^{2})=R_{k,\max}$, and $P_{t,\min}=P^{c}$. Therefore, $\alpha_{\max}=R_{k,\max}/P_{t,\min}$, and $\alpha =\left[\alpha_{\min},\alpha_{\max}\right]=\left[0,\sum_{k=1}^{K}R_{k,\max}/P^{c}\right]$.

### 4.2. Decentralized Algorithm for Subproblem $P_{3}$

The key step for finding the quantized noises and the beamformings in Algorithm 1 lies in solving the subproblem $P_{3}$. The main difficulty arises from the non-convex $R_{k}$ in the objective function (16a). Fortunately, by extending the equivalence between the SRmax problem and MMSE problem [31,35], $R_{k}$ in problem $P_{3}$ can be reformulated into a tractable form.

(17) $$R_{k}=\max_{\left\{\rho_{k},u_{k}\right\}}\;\frac{1}{\ln2}\left(\ln\rho_{k}-\rho_{k}e_{k}\right),$$

where $\rho_{k}\in R$ is a scalar variable associated with MU *k*, $e_{k}\in R$ is the MSE for MU *k*, given by

(18) ek=E|ukHyk−sk|2=|uk|2∑j=1K|hkwj|2+σ2+∑l=1Lql|hkl|2−2Re{ukhkHwk}+1.

The proof of the equality in (17) is based on the first-order optimality condition [31], which is omitted here for brevity. Then, problem $P_{3}$ can be recast as

(19) $$\begin{array}{cc} P_{4}:\min_{w,q}\min_{\rho ,u} & \;\frac{1}{\ln2}\sum_{k=1}^{K}\left(\rho_{k}e_{k}-\ln\rho_{k}\right)+\alpha P_{tot}\\ s.t. & \;(7b),(7c), \end{array}$$

where $u=[u_{1},\ldots ,u_{K}]$ and $\rho =[\rho_{1},\ldots ,u_{L}]$.

It is worth noting that problem $P_{4}$ is not jointly convex in {w,u,q,ρ}, but it is convex with respect to {w,q} or {u,ρ} by given {u,ρ} or {w,q}, respectively. Thus, with fixed $\left\{w_{k}\right\}$ and $\left\{q_{l}\right\}$, the optimal weight $\rho_{k}$ is $\rho_{k}=1/e_{k}^{⋆}$ where $e_{k}^{⋆}$ is the optimal MSE for MU *k*. Then, the optimal receive beamforming coefficient $\left\{u_{k}\right\}$ under fixed $\left\{w_{k}\right\}$, $\left\{q_{l}\right\}$ and $\left\{\rho_{k}\right\}$ is a MMSE receiver [31]

(20) $$u_{k}={\left(\sum_{j=1}^{K}{\left|h_{k}^{H}w_{j}\right|}^{2}+\sigma^{2}+\sum_{l=1}^{L}q_{l}{\left|h_{kl}\right|}^{2}\right)}^{-1}h_{k}^{H}w_{k}.$$

With fixed $\left\{u_{k}\right\}$ and $\left\{\rho_{k}\right\}$, the optimal $\left\{w_{k}\right\}$ and $\left\{q_{l}\right\}$ can be obtained by solving the following quadratic constraint quadratic programming (QCQP) problem.

(21) $$\begin{array}{cc} P_{5}:\min_{w,q} & \;f\left(w_{k}\right)+\sum_{k=1}^{K}\rho_{k}{\left|u_{k}\right|}^{2}\sum_{l=1}^{L}q_{l}{\left|h_{kl}\right|}^{2}+\frac{\alpha }{\tilde{\eta }}\sum_{l=1}^{L}p_{l}\\ s.t. & \;(7b),(7c), \end{array}$$

where f(wk)=∑k=1KwkHAwk−Re{bkHwk} is a quadratic objective function, and $b_{k}=2\rho_{k}u_{k}h_{k}$ and $A=\sum_{j=1}^{K}\rho_{j}{\left|u_{j}\right|}^{2}h_{j}h_{j}^{H}$, and $\tilde{\eta }=\eta \ln2$. It is observed that this problem is a convex optimization problem with respect to w and q, which can be solved centrally by standard mathematical tools, i.e., CVX [36]. It is noted that by simply replacing the constraints of the SINR and the maximum transmit power per BS as in [20], such an alternative optimization can also be adopted to solve $P_{0}$ with multivariate compression. That because the replacement does not affect the convexity of the subproblems. However, such an interior-point method solves $P_{5}$ with high computational complexity and it does not reveal the structure of the solution. Meanwhile, it is implementation intensive for large-scale C-RANs due to the centralized computation of the beamformings and quantization noises at the CU. Unlike the beamforming design problem in multicell system [30], the beamformings are coupled among BSs in our problem, making the Lagrangian based decomposition algorithm invalid in solving $P_{5}$. Towards this end, we propose a novel approach using ADMM method to solve $P_{5}$ with closed-form solution optimally in a parallel manner.

In particular, in $P_{5}$, the two constraints (7b) and (7c), respectively, provide an upper and lower bound on q. Then, the constraint $\beta_{l}p_{l}^{t}\leq P_{l}^{\max}-p_{l}^{t}$ should be satisfied. By rearranging this constraint, we have

(22) $$\left(1+\beta_{l}\right)p_{l}^{t}\leq P_{l}^{\max},\forall l.$$

Since the objective function of $P_{5}$ is monotonically decreasing over q, we can replace the inequality constraint (7c) with equality, i.e., $q_{l}=\beta_{l}\sum_{k=1}^{K}{\left|w_{kl}\right|}^{2}$. We denote a new beamforming vector for BS *l* as ${\tilde{w}}_{l}={\left[w_{1l},\ldots ,w_{Kl}\right]}^{T}$, and then we have $q_{l}=\beta_{l}{\left|{\tilde{w}}_{l}\right|}^{2}$. As a result, problem $P_{5}$ is equivalent to the following problem in only a single set of variables w.

(23a) $$\begin{array}{cc} P_{6}:\min_{w} & \;f\left(w_{k}\right)+\sum_{l=1}^{L}\mu_{l}{\left|{\tilde{w}}_{l}\right|}^{2} \end{array}$$

(23b) $$\begin{array}{cc} s.t. & \;\left(1+\beta_{l}\right){\left|{\tilde{w}}_{l}\right|}^{2}\leq P_{l}^{\max},\forall l, \end{array}$$

where $\mu_{l}=\sum_{k=1}^{K}\rho_{k}|u_{k}{|}^{2}\beta_{l}{\left|h_{kl}\right|}^{2}+\frac{\alpha }{\tilde{\eta }}\left(1+\beta_{l}\right)$.

The objective function in (23a) contains two parts, and they are functions of different variables, i.e., $w_{k}$ and ${\tilde{w}}_{l}$, rather than the same variable. Therefore, problem $P_{6}$ is not a standard group lest absolute shrinkage and selection operator (LASSO) problem [37]. Hence, the existing algorithms for the group LASSO problems are not directly applicable. This fact motivates us to find new algorithm to solve problem $P_{6}$. Fortunately, it is observed that problem $P_{6}$ has a special structure that can be solved by developing the famous ADMM algorithm. To account for the difference between $w_{k}$ and ${\tilde{w}}_{l}$ in problem $P_{6}$, we first introduce a copy ${\tilde{z}}_{l}$ for ${\tilde{w}}_{l}$, and define $z={\left[{\tilde{z}}_{1}^{T},\ldots ,{\tilde{z}}_{L}^{T}\right]}^{T}$. Problem $P_{6}$ can be equivalently expressed as

(24a) $$\begin{array}{cc} P_{7}:\min_{w,z} & \;f\left(w_{k}\right)+\sum_{l=1}^{L}\mu_{l}{\left|{\tilde{z}}_{l}\right|}^{2} \end{array}$$

(24b) $$\begin{array}{cc} s.t. & \;\left(1+\beta_{l}\right){\left|{\tilde{z}}_{l}\right|}^{2}\leq P_{l}^{\max},\forall l \end{array}$$

(24c) $$\begin{array}{c} \;{\tilde{z}}_{l}={\tilde{w}}_{l},\forall l. \end{array}$$

The partial augmented Lagrangian function of problem $P_{7}$ is

(25) L(w,z,y)=f(wk)+∑l=1Lμl|z˜l|2+∑l=1LRey˜lH(z˜l−w˜l)+c2∑l=1L|z˜l−w˜l|2,

where $y={\left[{\tilde{y}}_{1}^{T},\ldots ,{\tilde{y}}_{L}^{T}\right]}^{T}$ with ${\tilde{y}}_{k}={\left[{\tilde{y}}_{k1}^{T},\ldots ,{\tilde{y}}_{kL}^{T}\right]}^{T}$ is the vector of Lagrangian dual variables for the equality constraint (24c), and c>0 is some constant.

The idea of the ADMM is to update the local variables when fixing the other variables. Specifically, the variables updating procedure of the ADMM algorithm is detailed as follows.

By fixing $w^{\left(m\right)}$ and $y^{\left(m\right)}$ at the (m)-th iteration, $z^{\left(m+1\right)}$ at the (m+1)-th iteration is updated by solving the following convex problem

(26) $$\min_{z}\;L\left(w^{\left(m\right)},z,y^{\left(m\right)}\right)\;s.t.\;\left(24b\right).$$

We show that problem (26) can be solved in a parallel manner. Specifically, we first decompose problem (26) into *L* subproblems

(27) minz˜lμl|z˜l|2+Rey˜lH(z˜l−w˜l(m))+c2|z˜l−w˜l(m)|2s.t.(24b),

that can be solved independently at the CU.

The Karush-Kuhn-Tucker (KKT) conditions of problem (27) are

(28) $$\left(2\mu_{l}+2\theta_{l}^{⋆}+c\right){\tilde{z}}_{l}^{⋆}-cs_{l}=0,$$

(29) $$\left(1+\beta_{l}\right){\left|{\tilde{z}}_{l}^{⋆}\right|}^{2}\leq P_{l}^{\max},\theta_{l}^{⋆}\geq 0,$$

(30) $$\left(\left(1+\beta_{l}\right){\left|{\tilde{z}}_{l}^{⋆}\right|}^{2}-P_{l}^{\max}\right)\theta_{l}^{⋆}=0,$$

where $\theta_{l}^{⋆}$ is the optimal Lagrangian multiplier associated with the power constraint, ${\tilde{z}}_{l}^{⋆}$ is the optimum of ${\tilde{z}}_{l}$, and $s_{l}$ is defined as $s_{l}={\tilde{w}}_{l}^{\left(m\right)}-{\tilde{y}}_{l}^{\left(m\right)}$.

If $\theta_{l}^{⋆}=0$, we have ${\tilde{z}}_{l}^{⋆}=\frac{cs_{l}}{2\mu_{l}+c}$ under the condition of $\left(1+\beta_{l}\right){\left|{\tilde{z}}_{l}^{⋆}\right|}^{2}\leq P_{l}^{\max}$ (equivalent to $|s_{l}|\leq \sqrt{\frac{P_{l}^{\max}}{1+\beta_{l}}}\left(2\mu_{l}/c+1\right)$). If $\theta_{l}^{⋆}>0$, $\left(1+\beta_{l}\right){\left|{\tilde{z}}_{l}^{⋆}\right|}^{2}=P_{l}^{\max}$. Then, ${\tilde{z}}_{l}^{⋆}=\sqrt{\frac{P_{l}^{\max}}{1+\beta_{l}}}$ when $\left|s_{l}\right|>\sqrt{\frac{P_{l}^{\max}}{1+\beta_{l}}}\left(2\mu_{l}/c+1\right)$. Specially, when $|s_{l}|=0$, ${\tilde{z}}_{l}^{⋆}=0$. Therefore, $z_{l}^{\left(m+1\right)}={\tilde{z}}_{l}^{⋆}$ is updated as

(31) $$z_{l}^{\left(m+1\right)}=\left\{\begin{array}{cc} 0, & |s_{l}|=0\\ \frac{cs_{l}}{2\mu_{l}+c}, & \left|s_{l}\right|\leq \sqrt{\frac{P_{l}^{\max}}{1+\beta_{l}}}\left(2\mu_{l}/c+1\right)\\ \sqrt{\frac{P_{l}^{\max}}{1+\beta_{l}}}, & \left|s_{l}\right|>\sqrt{\frac{P_{l}^{\max}}{1+\beta_{l}}}\left(2\mu_{l}/c+1\right). \end{array}\right.$$

To update $w^{\left(m+1\right)}$ with fixed $\left\{y_{k}^{\left(m\right)},z_{k}^{\left(m+1\right)}\right\}$, the following problem is solved

(32) $$\min_{w}\;L\left(w,z^{\left(m+1\right)},y^{\left(m\right)}\right).$$

Due to the relationship between $w_{k}={\left[w_{kl},\ldots ,w_{kL}\right]}^{T}$ and ${\tilde{w}}_{l}={\left[w_{1l},\ldots ,w_{Kl}\right]}^{T},\forall l,k$, we have $\sum_{l=1}^{L}|{\tilde{w}}_{l}-{\tilde{z}}_{l}^{\left(m+1\right)}-{\tilde{y}}_{l}^{\left(m\right)}{/c|}^{2}=\sum_{k=1}^{K}{\left|w_{k}-z_{k}^{\left(m+1\right)}-y_{k}^{\left(m\right)}/c\right|}^{2}$. Problem (32) is equivalent to

(33) $$\min_{w}f\left(w_{k}\right)+\frac{c}{2}\sum_{k=1}^{K}{\left|w_{k}-z_{k}^{\left(m+1\right)}-y_{k}^{\left(m\right)}/c\right|}^{2}.$$

Then, problem (33) can be decomposed into the following *K* subproblems, and can be solved in a parallel manner with each MU.

(34) minwkwkHAwk−Re{bkHwk}+c2|wk−zk(m+1)−yk(m)/c|2.

By differentiating (34) with respect to $\left\{w_{k}\right\}$ and set to zero, we obtain the optimal $\left\{w_{k}^{⋆}\right\}$ with closed-form expression in the (m+1)-th iteration, given by

(35) $$w_{k}^{\left(m+1\right)}=w_{k}^{⋆}={\left(2A_{k}+cI\right)}^{-1}\left(2b_{k}+cz_{k}^{\left(m+1\right)}+y_{k}^{\left(m\right)}\right).$$

Using the relationship between $w_{k}$ and ${\tilde{w}}_{l}$, ${\tilde{w}}_{l}^{\left(m+1\right)}$ is obtained. Then, with obtained ${\tilde{w}}_{l}^{\left(m+1\right)}$ and ${\tilde{z}}_{l}^{\left(m+1\right)}$, the multipliers $y^{\left(m+1\right)}$ are updated

(36) $${\tilde{y}}_{l}^{\left(m+1\right)}={\tilde{y}}_{l}^{\left(m\right)}+c\left({\tilde{z}}_{l}^{\left(m+1\right)}-{\tilde{w}}_{l}^{\left(m+1\right)}\right);$$

Therefore, the decentralized algorithm for solving $P_{3}$ is summarized in Algorithm 3. The convergence of Algorithm 3 is guaranteed by Theorem 1.

**Algorithm 3** Decentralized Algorithm for Subproblem $P_{3}$. 1: Initialization: choose initial value for $w^{\left(0\right)}$ and $q^{\left(0\right)}$, set iteration index n=0, and choose the initial value for $z^{\left(0\right)}$ and $y^{\left(0\right)}$. 2: **Repeat** 3:    Update {u} by (20), and obtain $\left\{u^{\left(n\right)}\right\}$. 4:    Calculate $\left\{e_{k}\right\}$ according to (18) with $\left\{w^{\left(n\right)},q^{\left(n\right)}\right\}$. 5:    Update $\left\{\rho_{k}\right\}$ with $\rho_{k}=1/e_{k}$, and obtain $\rho^{\left(n\right)}$. 6:    Let iteration index m=0 and $w^{\left(m\right)}=w^{\left(n\right)}$. 7:    **while**
$\epsilon_{ADMM}\geq 10^{-5}$
**do** 8:       Update $z^{\left(m+1\right)}$ by (31); 9:       Update $w^{\left(m+1\right)}$ by (35); 10:       Update the multipliers $y^{\left(m+1\right)}$ by (36). 11:        m=m+1. 12:    **end while** 13:     n=n+1. 14:    Update $w^{\left(n\right)}=w^{\left(m+1\right)}$. 15:    Update $\left\{q^{\left(n\right)}\right\}$ by $q_{l}=\beta_{l}\sum_{k=1}^{K}{\left|w_{kl}\right|}^{2},\forall l$ with $w^{\left(n\right)}$. 16: **Until** convergence or the maximum iteration number is reached.

**Theorem** **1.** *Algorithm 3 generates a sequence $\left\{w^{\left(n\right)},q^{\left(n\right)}\right\}$ that converge to a stationary point of problem $P_{3}$.*

**Proof.**  The proof is based on the convergence of the alternative optimization method and ADMM algorithm. With initialized $\left\{w^{\left(0\right)},z^{\left(0\right)},y^{\left(0\right)}\right\}$, the inner loop of Algorithm 3 from steps 7 to 12 converges to an optimal solution of $P_{5}$ due to the convergence of ADMM algorithm, and the proof can be found in [38]. On the other hand, the outer loop of Algorithm 3 converges to a stationary point of subproblem $P_{3}$ due to the convergence property of block coordinate decent algorithm [31,35]. According to [25,30], for an arbitrary α, the objective (16a) in problem $P_{3}$ is shown to be non-decreasing during each iteration of the outer loop of Algorithm 3. Therefore, Algorithm 3 is guaranteed to converge to a stationary point of problem $P_{3}$, and the proof is completed.  ☐

Denote by $\alpha^{\mathrm{opt}}$ the actually optimal solution of problem $P_{0}$, and $\alpha^{⋆}$ the obtained suboptimal solution returned by the Algorithm 2 when using Algorithm 3 to solve problem $P_{3}$. Since Algorithm 3 converges to a stationary point of problem $P_{3}$, the suboptimal objective of G(α) equals to zero which equivalently equals to $G(\alpha^{⋆})>0$. Moreover, considering the fact that G(α) is monotonically decreasing, the actually optimal solution $\alpha^{\mathrm{opt}}$ that satisfies $G(\alpha^{\mathrm{opt}})=0$ must be no smaller than $\alpha^{⋆}$, i.e., $\alpha^{\mathrm{opt}}\geq \alpha^{⋆}$. Therefore, the obtained solution returned by Algorithm 2 when using Algorithm 3 is no larger than the optimal solution of problem $P_{0}$. Simulation results will demonstrate that the obtained solution is very close to the optimal one that verifies the effectiveness of the proposed algorithm numerically.

Combining Algorithms 2 and 3, it is concluded that problem $P_{0}$ can be solved efficiently by the proposed TLD algorithm. Since the limits of the upper and lower bounds are updated iteratively, the bisection procedure will stop any way. However, we may not have G(α)=0.

#### 4.2.1. Parallelized Implementation

Since problem (26) is decomposed into *L* subproblems, $z^{\left(m+1\right)}$ at step 8 in Algorithm 3 is updated in a parallel manner with closed-form solution. Similarly, the beamformings $w^{\left(m+1\right)}$ and multipliers $y^{\left(m+1\right)}$ are also updated using (35) and (36) in a parallel manner with closed-form solution. Therefore, we derive closed-form expressions for the optimal beamformings, the optimal receiver filters and the auxiliary variables in Algorithm 3, that provide some insights on the EEmax problem. For example, if $w_{l}=0$ (no transmission power consumed by BS *l*), BS *l* can be switched off to save static power and improve EE.

#### 4.2.2. Complexity Analysis

According to the algorithm flow, the computational complexity of Algorithm 3 contains two parts. In Algorithms 3, the computational complexity of computing (20) arises from the matrix inversion of the receive beamforming, i.e., $O(L^{3})$. When the interior-point method is adopted to solve $P_{5}$, the computational complexity is $O({\left(LK\right)}^{3.5})$ [39]. In this case, to serve *K* MUs, the overall computational complexity is in the order of $O(KL^{3}+{\left(LK\right)}^{3.5})$. On the other hand, since the ADMM algorithm is applied to solve $P_{5}$, the main computational complexity is the matrix inversion in step 9 of Algorithm 3 where the transmit beamformings are computed by (35). Thus, the overall computational complexity of Algorithm 3 is in the order of $O(KL^{3}+KL^{3})$. For the outer layer algorithm, simulation results will show that it converges rapidly (about 5 iterations). Therefore, we can deduce that the proposed TLD algorithm is computation efficient.

#### 4.2.3. Generalization to the Multi-Antenna System

Although a single-antenna is equipped at each BS and each MU in the above discussion, we claim that proposed TLD algorithm can be generalized to the multi-antennas BSs and the single-antenna MUs scenarios. This is because deployed multiple antennas (each with *N* antennas) at BSs, one only needs to replace the corresponding channel coefficient $h_{kl}$ from BS *l* to MU *k* with $h_{kl}\in C^{N\times 1}$. Similarly, the transmit beamforming coefficient $w_{kl}$ and receive beamforming coefficient $u_{k}$ from BS *l* to MU *k* are replaced with vectors $w_{kl}\in C^{N\times 1}$ and $u_{k}\in C^{N\times 1}$, respectively. While $e_{l}$ is replaced by $e_{l}\in C^{N\times 1}$, and the corresponding covariance matrice is $E\left[e_{l}e_{l}^{H}\right]=\mathrm{diag}\left(q_{11},\ldots ,q_{1N}\right)$. If $q_{l}=q_{11}=\ldots =q_{lN}$, one only need to replace $q_{l}$ in (7b) and (7c) with $Nq_{l}$. Therefore, the proposed TLD algorithm can be extended to solve the EEmax problem with multi-antenna BSs and single-antenna MUs but requires additional efforts. Moreover, for the multi-antenna BSs and multi-antenna MUs C-RAN, we point out that the weighted minimum MSE algorithm in [31] might provide some insights on how to apply the proposed algorithm.

## 5. Robust Algorithms with Imperfect CSI

In practical C-RAN, due to the limited feedback [40], partial CSI [41] or estimated error, the obtained channel is not perfect. According to [42,43,44], the imperfection in CSI has significantly impact on the system performance. Therefore, we will extend the proposed algorithms in the previous two sections to solve the robust EEmax problem in the presence of imperfect CSI.

The same system model is considered as in Section 2. Since the path-loss fading and the log-normal fading can be estimated accurately, the imperfection usually comes from the uncertain small-scale fading. Thus, different from the worst-case design [42,43], the Gaussian distribution in [44] is adopted to model the channel imperfection. The real channel from BS *l* to MU *k* is expressed as

(37) $$h_{kl}=g_{kl}\left({\tilde{h}}_{kl}+{\tilde{\Delta }}_{kl}\right),\forall l,k,$$

where $g_{kl}=GL\left(d_{kl}\right)\varphi_{kl}$ is the channel gain consisting of the antenna gain *G*, the path-loss fading $L(d_{kl})$ at distance $d_{kl}$ in km and the log-normal random fading $\varphi_{kl}$. ${\tilde{h}}_{kl}$ and ${\tilde{\Delta }}_{kl}$ are respectively the estimated channel and channel uncertainty from BS *l* to MU *k*. ${\tilde{\Delta }}_{kl}$ is assumed to be independent identically distributed (i.i.d) zero mean circularly symmetrical complex Gaussian (ZMCSCG) random variables with variance $\sigma_{e}^{2}$. The channel from all the BSs to MU *k* is $h_{k}=g_{k}\left({\tilde{h}}_{k}+{\tilde{\Delta }}_{k}\right),\forall k,$ where $g_{k}=\mathrm{diag}\left(g_{kl},\ldots ,g_{kL}\right)$, and ${\tilde{h}}_{k}$ and ${\tilde{\Delta }}_{k}$ are the aggregated collection of ${\tilde{h}}_{kl}$ and ${\tilde{\Delta }}_{kl}$, respectively. Let ${\bar{h}}_{k}=g_{k}{\tilde{h}}_{k}$, ${\bar{h}}_{kl}=g_{kl}{\tilde{h}}_{kl}$ and $D_{k}=\mathrm{diag}(|g_{k1}{|}^{2},\ldots ,|g_{kL}{|}^{2})$. The received SINR at MU *k* is expressed as

(38) $$\begin{array}{c} {\mathrm{SINR}}_{k}=\frac{|{\bar{h}}_{k}^{H}w_{k}{|}^{2}}{\sum_{i\neq k}|{\bar{h}}_{k}^{H}w_{i}{|}^{2}+\sigma^{2}+\sum_{l=1}^{L}q_{l}\left(\right|{\bar{h}}_{kl}{|}^{2})+Y}, \end{array}$$

where $Y=\sigma_{e}^{2}\mathrm{Tr}\left(D_{k}\right)\sum_{i=1}^{K}{\left|w_{i}\right|}^{2}+\sum_{l=1}^{L}q_{l}\sigma_{e}^{2}$ denotes the interference caused by uncertainty part of CSI. The first term of Y contains the interference caused by the CSI error of the intended signal and the signals of other MUs.

The robust EEmax problem has the same form as problem $P_{0}$ by simply using the imperfect channel, i.e., replacing $h_{kl}$ by (28). The details of the extended algorithms to solve the robust problem with the consideration of imperfect CSI are given in the following two subsections, respectively.

### 5.1. Robust Optimal Algorithm

To apply the optimal algorithm under imperfect CSI, we only need to reformulate (8b) because the CSI is only involved in (8b). In particular, (8b) is recast as

(39a) $$\begin{array}{c} \frac{1}{{\tilde{\gamma }}_{k}}{\bar{h}}_{k}w_{k}\geq {\left(\sum_{i\neq k}{\left|{\bar{h}}_{k}^{H}w_{i}\right|}^{2}+\sigma^{2}+\sum_{l=1}^{L}q_{l}\left(\right|{\bar{h}}_{kl}{|}^{2})+Y\right)}^{1/2}, \end{array}$$

(39b) Im(hkwk)=0,∀k∈K.

Follow the same procedure as demonstrated in Algorithm 1, one only need to replace constraint (10a) in problem $P_{2}$ with (39a). In this case, the computational complexity is the same as Algorithm 1 (both of them are very high but provide optimal solutions).

### 5.2. Robust TLD Algorithm

Since the CSI is involved only in $R_{k}$, the TLD algorithm performs well to solve the robust EEmax problem but requires some transformations. In particular, the out layer iteration of the robust TLD algorithm follows the procedure as Algorithm 2. To solve the robust subproblem $P_{3}$ in the inner layer, Algorithm 3 cannot be applied directly. Fortunately, with the same transformations as Section 4.2, the corresponding MMSE receiver can be expressed as

(40) $$\begin{array}{c} {\tilde{u}}_{k}=\frac{{\bar{h}}_{k}^{H}w_{k}}{\sum_{j=1}^{K}|{\bar{h}}_{k}^{H}w_{i}{|}^{2}+\sigma^{2}+\sum_{l=1}^{L}q_{l}\left(\right|{\bar{h}}_{kl}{|}^{2})+Y}, \end{array}$$

and the MSE of the *k*-th MU is

(41) e˜k=E|u˜kHyk−sk|2=|u˜k|2∑j=1KTrwj(h¯kh¯kH)wjH+|u˜k|2σ2+1−2Re{u˜kh¯kHwk}+|u˜k|2(∑l=1Lql|h¯kl|2+Y).

Then, with fixed $\left\{{\tilde{u}}_{k}\right\}$ and $\left\{\rho_{k}\right\}$, the robust $P_{5}$ with respect to $\left\{w_{k}\right\}$ and $\left\{q_{l}\right\}$ becomes

(42) $$\begin{array}{cc} P_{8}:\min_{w,q} & \;\sum_{k=1}^{K}\rho_{k}{\tilde{e}}_{k}+\frac{\alpha }{\tilde{\eta }}\sum_{l=1}^{L}p_{l}\\ s.t. & \;(6b),(6c). \end{array}$$

We further reformulate problem $P_{8}$ as

(43a) $$\begin{array}{cc} P_{9}:\min_{w} & \;\tilde{f}\left(w_{k}\right)+\sum_{l=1}^{L}{\tilde{\mu }}_{l}{\left|{\tilde{w}}_{l}\right|}^{2} \end{array}$$

(43b) $$\begin{array}{cc} s.t. & \;\left(1+\beta_{l}\right){\left|{\tilde{w}}_{l}\right|}^{2}\leq P_{l}^{\max},\forall l, \end{array}$$

where f˜(wk)=∑k=1KwkHA˜wk−Re{b˜kHwk}, ${\tilde{\mu }}_{l}=\sum_{k=1}^{K}\rho_{k}|{\tilde{u}}_{k}{|}^{2}\beta_{l}\left(\right|{\bar{h}}_{kl}{|}^{2}+\sigma_{e}^{2})+\frac{\alpha }{\tilde{\eta }}\left(1+\beta_{l}\right)$, and

(44) $$\tilde{A}=\sum_{j=1}^{K}\rho_{j}{\left|{\tilde{u}}_{j}\right|}^{2}\left({\bar{h}}_{j}{\bar{h}}_{j}^{H}+\sigma_{e}^{2}D_{j}\right),$$

(45) $${\tilde{b}}_{k}=2\rho_{k}{\tilde{u}}_{k}{\bar{h}}_{k}.$$

Since problems $P_{6}$ and $P_{9}$ have the same form, the ADMM algorithm presented in the inner layer of Algorithm 3 performs well in solving $P_{9}$. Particularly, z is updated by (31), and $\left\{w_{k}\right\}$ is updated using the following closed-form expression

(46) $$w_{k}={\left(2{\tilde{A}}_{k}+cI\right)}^{-1}\left(2{\tilde{b}}_{k}+cz_{k}+y_{k}\right),$$

where ${\tilde{A}}_{k}$ and ${\tilde{b}}_{k}$ are shown in (44) and (45), respectively.

The corresponding multipliers y are updated by (36). Similar to the TLD algorithm, the robust TLD algorithm procedure is omitted here for brevity. It is easily verified that the robust TLD algorithm is convergent, and it has the same computational complexity as the non-robust TLD algorithm, and it achieves a suboptimal solution with closed-form expression in a parallel manner as well.

## 6. Simulation Results and Discussions

**Figure 1:**
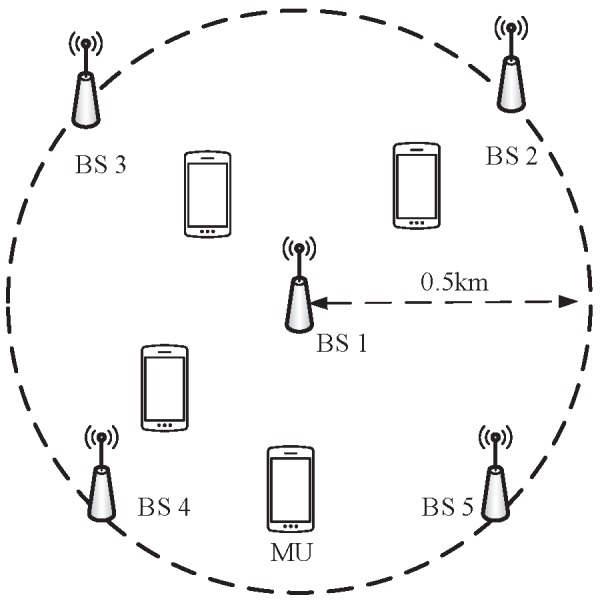
Simulation setup in a downlink C-RAN.

In this section, we evaluate the performance of the proposed algorithms via Monte-Carlo simulation. We consider a downlink C-RAN with L=5 single-antenna BSs and K=4 single-antenna MUs, where one BS locates at the circle centre, and the other four BSs are located in a circle region at equal distances apart with radius 0.5 km, as shown in Figure 1. The four single-antenna MUs are randomly deployed in the circle with uniform distribution. Each BS and MU are equipped with a single antenna, and we set the maximum transmit power $P^{m}=P_{l}^{\max}$ and the fronthaul capacity $C=C_{l}$ for all BSs. The convergence errors are set as ϵ for all the proposed algorithms. Unless specified, other simulation parameters are listed in Table 1, and all the simulation results are averaged over 50 times independent MU locations, each with a single random channel realization. In order to verify the effectiveness of the proposed algorithms, we consider two baseline algorithms for comparison:

**SRmax algorithm**: The proposed TLD algorithm is adopted to solve the SRmax problem by simply setting α=0.

**DC algorithm**: We also modify the algorithm proposed in [22] for comparison. Specifically, to arrive at a tractable formulation, one can use the epigraph form of the original problem. Based on the transformation, one can transform the constraints into convex ones by using the first-order Taylor expansion. Then, the approximated problem is iteratively solved, and the solution converges to a KKT point [22]. The computational complexity of the DC algorithm using the interior-point method is $O(I_{\max}L^{6.5}K^{3.5}N^{6.5})$ ($I_{\max}$ is the maximum of iteration number), which is much higher than the TLD algorithm.

### 6.1. Non-Robust Performance

**Figure 2:**
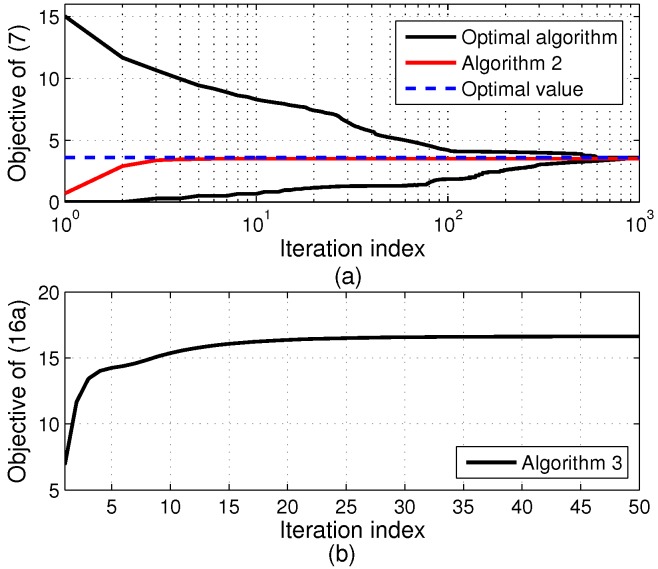
Convergence of the proposed algorithms over a typical random channel realization. (**a**) EE of Algorithms 1 and 2; (**b**) Objective of problem $P_{3}$ with α = 2.90 (at the 2nd iteration of Algorithm 2 in Figure 2a).

We first investigate the convergence behavior of the proposed algorithms over a typical random channel realization for $P^{c}$ = 2 W, $P^{m}$ = 30 dBm, and *C* = 5 bits/s/Hz, which are presented in Figure 2.

It is observed that the outer layer (Algorithm 2) of TLD in Figure 2a converges to a near optimum very fast (about 5 iterations). Meanwhile, Figure 2b illustrates that the objective function of problem (16) of Algorithm 3 at the 2nd iteration of Algorithm 2 in Figure 2a increases and converges to a stationary point in less than 30 iterations. Thus, the proposed TLD algorithm converges rapidly. It is also found that the convergence rate of the optimal algorithm is much slower that the TLD algorithm. Due to a large number of infeasible boxes being removed, the gap between upper and lower bounds of the optimal algorithm reduces rapidly during the first iterations.

**Figure 3:**
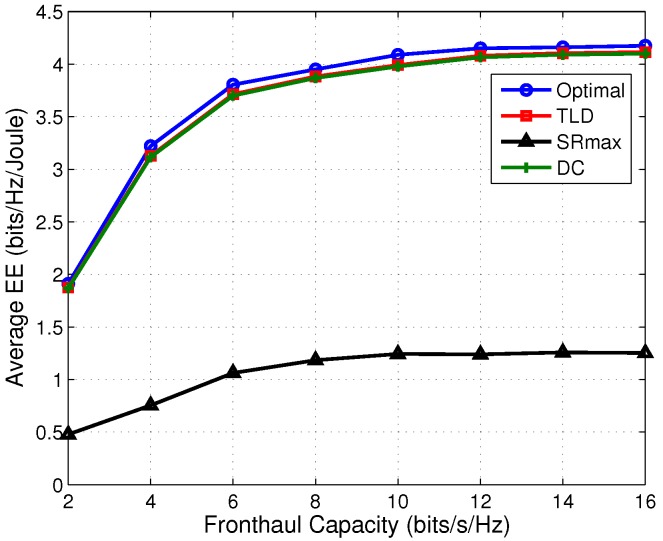
Average EE at various fronthaul capacities.

**Figure 4:**
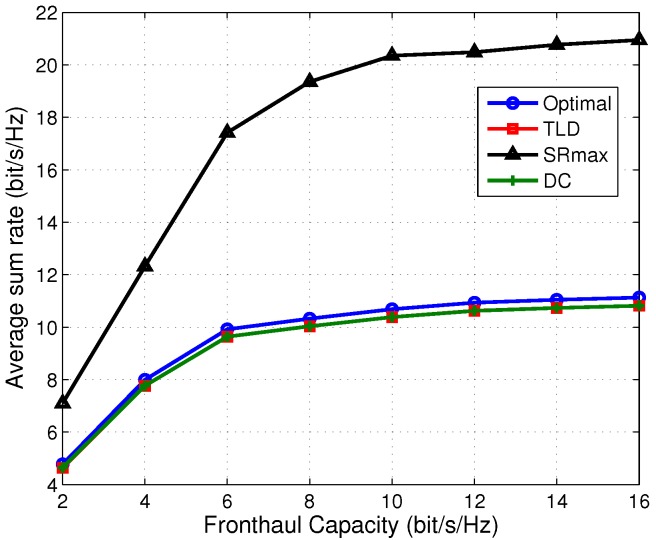
Average sum rate at various fronthaul capacities.

**Figure 5:**
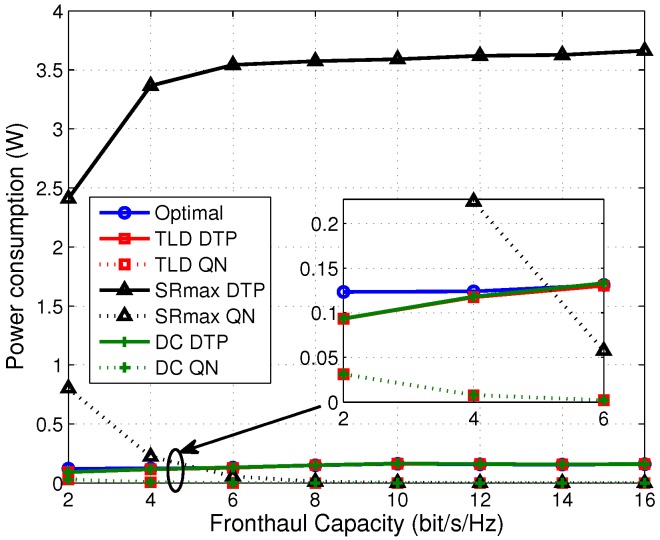
Average power consumption at various fronthaul capacities.

Then, we explore the effect of fronthaul capacity on the performance in terms of EE, sum rate and transmit power, shown in Figure 3, Figure 4 and Figure 5, respectively.

The TLD algorithm achieves a comparable performance compared to the DC one in terms of EE. Figure 3 shows the EE comparison among the four algorithms under different fronthaul capacities. As an be seen from Figure 3 that the TLD and DC algorithms achieve an approximate EE performance as the optimal one, and both of them outperform the SRmax one by about three times in the middle-high fronthaul capacity region. Since the network power is penalized in (7a), and the sum rate and power are jointly optimized, the EE is improved compared to SRmax. This can be explained from Figure 4 and Figure 5 that the saved ratio of the transmit power of TLD (save for about 95% compared with SRmax) is much higher than its sum rate reduction ratio (decrease by about 50% compared with SRmax), resulting in a higher EE. It is also observed from Figure 3 that the EE performance increases with the growing fronthaul capacity in the low fronthaul capacity region, and then gradually converge to a constant in the middle to high fronthaul capacity region, and a similar trend can be found in Figure 4. According to the expression of quantization noises (QN), i.e., $q_{l}=\frac{1}{2^{C}-1}\sum_{k=1}^{K}{\left|w_{kl}\right|}^{2}$, it reduces exponentially with the increasing fronthaul capacity (shown in Figure 5). As a result, at low fronthaul capacity region, the SINR is limited by the high QN, leading to a low sum rate in this region. Moreover, the corresponding data transmit power (DTP) in Figure 5 increases very slowly from low to high fronthaul capacity region. That because with the increase of the fronthaul capacity, more powers of DTP and QN are consumed to improve the sum rate. However, more network power is consumed which makes the increment of EE gradually. It should be noted that the optimal algorithm cannot provide DTP and QN but only the total network power, as a result only the transmit power (sum of DTP and QN) of the optimal algorithm is given in Figure 5. In summary, Figure 3, Figure 4 and Figure 5 illustrate that the TLD and optimal algorithms can achieve the balance between the sum rate and power consumption, implying a much higher EE than the SRmax one.

**Figure 6:**
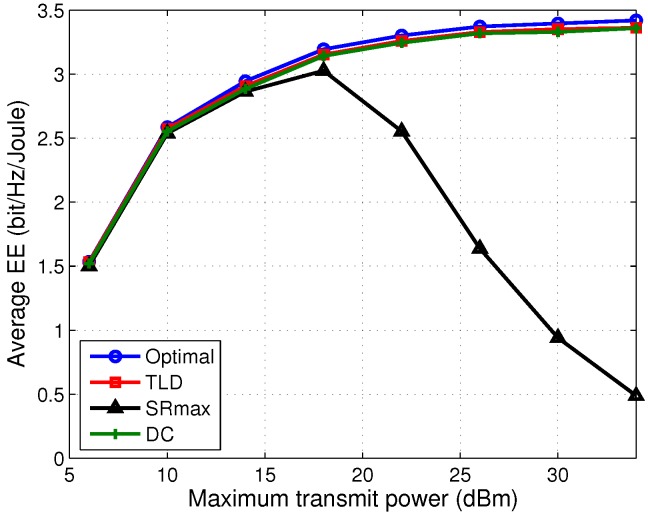
Average EE versus maximum transmit power with $P^{c}=2$ W and C=5 bits/s/Hz.

Figure 6 compares the EE performance with respect to the maximum transmit power $P^{m}$ for a given fronthaul capacity C=5 bits/s/Hz. It is observed that TLD and DC significantly outperform the SRmax in terms of EE in the middle to high transmit power region (≥18 dBm). This is because by jointly optimizing the sum rate and network power, the increased sum rate is slightly larger than the increased power consumption ratio, resulting in a gradually increase of EE. While for the SRmax algorithm, because of the interference between MUs, its sum rate gain cannot compensate for the negative impact of the network power, making its EE worse than the TLD and DC algorithms in the event that $P^{m}$≥ 18 dBm. It is also observed that the optimal, and TLD and DC algorithms have comparable EE performance as the SRmax one at low maximum transmit power (≤18 dBm), which suggests that at this region, transmitting with the maximum transmit power provides comparable sum rate gain for the optimal, EEmax and SRmax algorithms.

**Figure 7:**
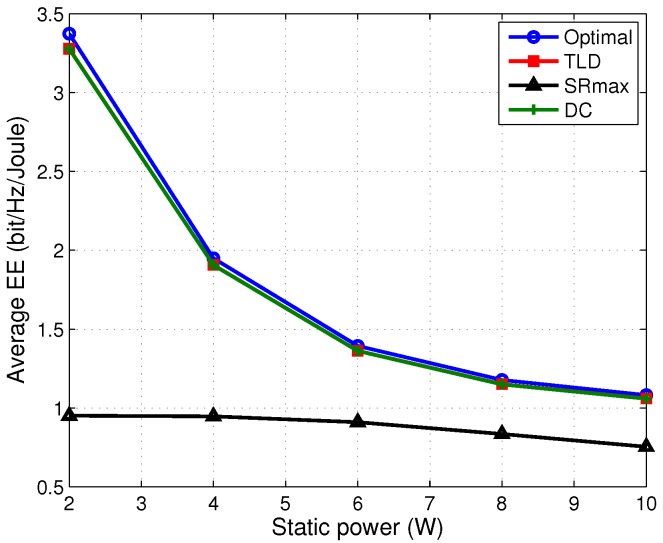
Average EE versus static power with $P^{m}=30$ dBm and C=5 bits/s/Hz.

To investigate further, we also test the impact of static power consumption on the EE performance of the proposed algorithms, shown in Figure 7. It depicts that, at low to middle static power ($P^{c}\leq 6$ W), the EE of the optimal, DC and TLD algorithms decreases significantly with the growing static power $P^{c}$. This can be explained that the static power dominates the network power at this region and the transmit power is optimized as well, leading to a rapid decrease of EE. Whereas, since the maximum transmit power is usually adopted by the SRmax algorithm for transmission and it accounts for a large amount of the network power for $P^{c}$ ranging from 2 to 10 W, the decrease EE of the SRmax algorithm becomes gentle. These facts bring a higher EE performance for the TLD algorithm than the SRmax at different static powers.

**Figure 8:**
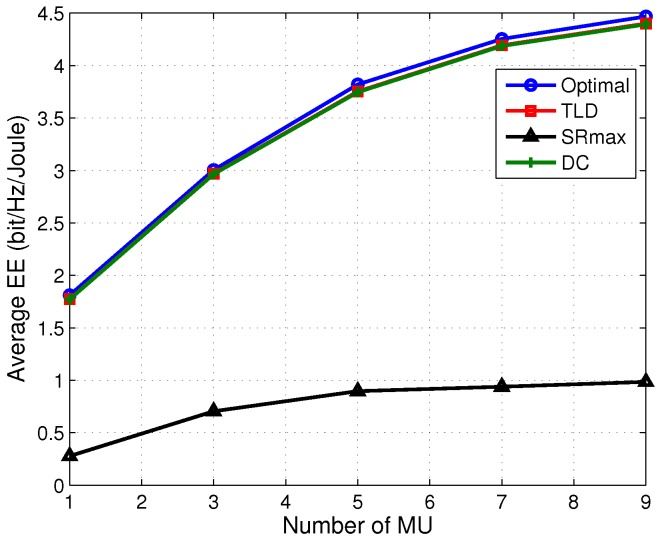
Average EE versus MU number with $P^{m}=30$ dBm, C=5 bits/s/Hz and $P^{c}=2$ W.

The effect of MU number on the EE performance is also investigated with C=5 bits/s/Hz, $P^{m}=30$ dBm and $P^{c}=2$ W, which is shown in Figure 8. We learn from Figure 8 that, with more MUs to be served, the EE of the TLD and DC algorithms increase significantly and exhibits obvious advantage over the SRmax one, e.g., higher than SRmax by more than 300%. Intuitively, the data rate of each MU decreases with the growing number of served MUs due to the increased interference between MUs. The obtained sum rate gain is very large when the serving MU number is small, while the sum rate increases much more slowly if the served MU number is large. It is attributed to the rapidly increase of interference among MUs by serving large number of MUs, implying a data rate reduction for each MU and a slightly increase on the sum rate of all MUs. Besides, more transmit power is needed to achieve the sum rate gain when serving more MUs, which also perform negative for boosting EE performance. It should also be noted that by further increasing the number of MUs, the EE of all the four algorithms will firstly become stable and then show a decrease trend. This is because, due to the increased interference caused by increasing the MU number, the decreased sum data rate of MUs is much larger than the increased data rate brought by the newly added MUs.

### 6.2. Robust Performance

**Figure 9:**
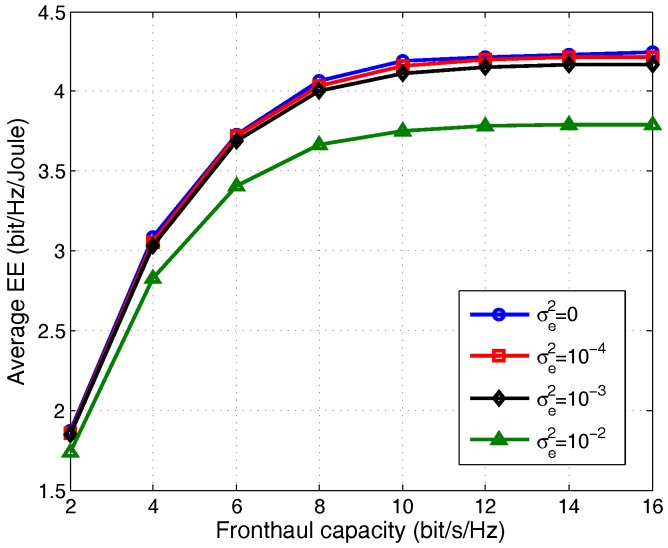
Average EE versus fronthaul capacity with different channel errors, $P^{m}=30$ dBm, $P^{c}=2$ W and C=5 bits/s/Hz.

The EE performance of the proposed robust TLD algorithm is investigated over the channels with different channel errors, and the results are shown in Figure 9. Similar to the non-robust design, each point in Figure 9 is averaged over 100 channel realizations. In the simulation, $\sigma_{e}^{2}=0$ means perfect CSI is known at the CU. It was observed in the previous subsection that the SRmax algorithm has a worse EE performance than that of the TLD (TLD has near optimal performance), and thus we only plot the EE curve of robust TLD algorithm under different CSI errors. Due to the high complexity imposed by the DC algorithm, we do not compare it with the TLD in this section.

We can see from Figure 9 that by increasing the channel errors from $\sigma_{e}^{2}=0$ to $\sigma_{e}^{2}=10^{-2}$, the EE performance decreases significantly especially in the middle to high fronthaul capacity. In the low fronthaul capacity region, according to (38), the received SINR is slightly influenced by the channel errors since the increased interference is very small. While a worse sum rate is obtained in the middle to high fronthaul capacity due to the impairment of channel error on the received SINR, and more amount of transmit power is consumed in order to achieve the same sum rate for a larger channel errors. As a result, the sum rate gain cannot compensate the network power reduction that caused by the channel error, resulting in lower EE. In summary, the EE performance is susceptible to the channel error especially for a larger channel error.

## 7. Conclusions

In this paper, we have studied the EEmax problem with fronthaul compression in a downlink C-RAN. The optimization problem is formulated as a non-convex fractional programming problem. We have proposed an optimal algorithm based on BnB method to provide a performance benchmark. Further, a near optimal TLD algorithm has been proposed via a bisection search procedure in conjunction with an ADMM method. The proposed algorithms were guaranteed to be convergent, and the solution of TLD was achieved with closed-form in a parallel manner. Simulation results illustrated that the TLD algorithm converged to a near optimal solution very fast, and it achieved a much higher EE than the SRmax algorithm. The results further indicated that EE could be improved by increasing the fronthaul link capacity or optimizing the network power. Numerical analysis also demonstrated that the robust TLD algorithm can provide robust performance in the case of lacking perfect CSI, and its performance is susceptible to the channel error. 

## Figures and Tables

**Table 1:** Simulation parameters.

Parameter	Value
Maximum transmit power $P^{m}$	1 W
Power amplifier efficiency η [4]	1/4
Transmit antenna power gain *G*	9 dBi
Background noise $\sigma^{2}$	−104 dBm
Path-loss fading from BS *l* to MU *k* [4]	148.1 + 37.6$\log_{10}d_{kl}$
Log-normal shadowing $\varphi_{kl}$	8 dB
Static power $P^{c}$	2 W
Convergence errors ϵ=$\epsilon_{ADMM}$	$10^{-5}$
Maximum iteration number	50
